# Role of SpO2/FiO2 Ratio and ROX Index in Predicting Early Invasive Mechanical Ventilation in COVID-19. A Pragmatic, Retrospective, Multi-Center Study

**DOI:** 10.3390/biomedicines9081036

**Published:** 2021-08-18

**Authors:** Ana Alberdi-Iglesias, Francisco Martín-Rodríguez, Guillermo Ortega Rabbione, Ana I. Rubio-Babiano, María G. Núñez-Toste, Ancor Sanz-García, Carlos del Pozo Vegas, Miguel A. Castro Villamor, José L. Martín-Conty, Cristina Jorge-Soto, Raúl López-Izquierdo

**Affiliations:** 1 Emergency Department, Valladolid University Clinical Hospital, Castilla y León Regional Health Management (SACYL), 47005 Valladolid, Spain; aalberdi@saludcastillayleon.es (A.A.-I.); airubio@saludcastillayleon.es (A.I.R.-B.); mgnunez@saludcastillayleon.es (M.G.N.-T.); cpozove@saludcastillayleon.es (C.d.P.V.); 2 Advanced Clinical Simulation Centre, Advanced Life Support Unit, Emergency Medical Services, Faculty of Medicine, Universidad de Valladolid, 47005 Valladolid, Spain; 3 Data Analysis Unit, Health Research Institute, Hospital de la Princesa, Madrid (IIS-IP), C/Diego de León, 62, 28006 Madrid, Spain; agetro.ortega@gmail.com (G.O.R.); ancor.sanz@gmail.com (A.S.-G.); 4 Centro de Simulación Clínica Avanzada, Facultad de Medicina, Universidad de Valladolid, 47005 Valladolid, Spain; mcastrovi@saludcastillayleon.es; 5 Faculty of Health Sciences, Universidad de Castilla la Mancha, 45600 Talavera de la Reina, Spain; JoseLuis.MartinConty@uclm.es; 6 Grupo de Investigación CLINURSID, Facultad de Enfermería, Universidad de Santiago de Compostela, 15782 Santiago de Compostela, Spain; cristina.jorge@usc.es; 7 Emergency Department, Hospital Universitario Rio Hortega de Valladolid, Gerencia Regional de Salud de Castilla y León (SACYL), c/Dulzaina, 2, 47012 Valladolid, Spain; rlopeziz@saludcastillayleon.es

**Keywords:** clinical decision making, COVID-19, emergency care, hypoxemia, mechanical ventilation, risk scores

## Abstract

The ability of COVID-19 to compromise the respiratory system has generated a substantial proportion of critically ill patients in need of invasive mechanical ventilation (IMV). The objective of this paper was to analyze the prognostic ability of the pulse oximetry saturation/fraction of inspired oxygen ratio (SpO2/FiO2) and the ratio of SpO2/FiO2 to the respiratory rate–ROX index–as predictors of IMV in an emergency department in confirmed COVID-19 patients. A multicenter, retrospective cohort study was carried out in four provinces of Spain between March and November 2020. The discriminative power of the predictive variable was assessed through a prediction model trained using a derivation sub-cohort and evaluated by the area under the curve (AUC) of the receiver operating characteristic (ROC) on the validation sub-cohort. A total of 2040 patients were included in the study. The IMV rate was 10.1%, with an in-hospital mortality rate of 35.3%. The performance of the SpO2/FiO2 ratio was better than the ROX index–AUC = 0.801 (95% CI 0.746–0.855) and AUC = 0.725 (95% CI 0.652–0.798), respectively. In fact, a direct comparison between AUCs resulted in significant differences (*p* = 0.001). SpO2 to FiO2 ratio is a simple and promising non-invasive tool for predicting risk of IMV in patients infected with COVID-19, and it is realizable in emergency departments.

## 1. Introduction

Coronavirus disease 2019 (COVID-19) has caused a major disruption in the normal operations of healthcare systems, demanding a reorientation of all routine care towards strategic medicine [1,2]. One of the primary challenges during the current pandemic has been the assessment of a large pool of patients affected by a new disease with no clear specific symptomatology and few treatment options, but accompanied by a non-proportional increase in resources or hospitals capacity [3]. The overall front-line emergency system has been dramatically altered, from family centers and emergency medical services to emergency departments (ED) [4,5].

COVID-19′s ability to compromise the respiratory system has generated a proportion of critically ill patients who develop novel severe acute respiratory syndrome coronavirus 2 (SARS-CoV-2) and who require invasive mechanical ventilation (IMV), intensive care unit admission, and who suffer from high associated mortality [6]. The identification of the so-called *silent hypoxemia*, characterized by a dissociation between oxygen saturation (SpO2) and ventilatory dynamics [7,8], has been strongly emphasized recently. Moreover, some studies have shown that delayed intubation may even worsen [9]. These observations highlight the need to identify objective measurements of early predictors of mechanical ventilation [10,11]. Under normal conditions, arterial blood gases can determine true oxygenation status but, in the current pandemic situation, non-invasive solutions are needed to guide actions as early as during hospital triage [12,13].

Typically, the ratio of SpO2/FiO2 (oxygen saturation measured by pulse oximetry/fraction of inspired oxygen) to respiratory rate—the ROX index—has been used as a predictor of high-flow nasal cannula failure/need for intubation [14]; however, its use has been extended to other clinical contexts, as is the case with COVID-19 [15,16]. As an alternative, the SpO2/FiO2 ratio has been proposed. Both can be performed at any time and under any clinical conditions, both continuously and non-invasively [17,18].

A study has suggested that the SpO2/FiO2 ratio could be used as a prognostic marker in the management of COVID-19 patients, with the objective of early improvement in the adjustment of treatments in the intensive care unit (ICU) [19]. It has also been evaluated as a possible index for triage upon admission of patients admitted for acute respiratory symptoms, particularly in the case of suspected COVID-19 [20]. Moreover, if respiratory rate is available, ROX index, (SpO2/FiO2)/respiratory rate) can also be obtained [15,21]; its implementation is an additional tool to help emergency services assess the clinical severity of COVID-19 patients, and it also serves as a safety measure during hospital discharges (10). It has even been shown to be potentially useful in guiding the decision to intubate COVID-19 patients, especially those with moderate to acute respiratory failure under non-ICU conditions [22].

The primary purpose of this study was to analyze the prognostic ability of both SpO2/FiO2 and the ROX indices in confirmed COVID-19 patients, during initial contact with ED, as predictors of prompt deterioration, as objectified by requirement IMV. The relationship between the performance of both parameters to the age of patients was also analyzed.

## 2. Materials and Methods

### 2.1. Study Design and Settings

A multicenter retrospective cohort study was carried out in the provinces of Palencia, Salamanca, Segovia, and Valladolid (Spain) between March and November 2020. The study involved ten advanced life support units, fifty-one basic life support units, and eight hospitals (three tertiary university hospitals, four general district hospitals, and one local hospital). All the facilities involved depend on the public health system—Sanidad de Castilla Y León (SACYL)—which is the major healthcare operator responsible for regional responses to the COVID-19 pandemic.

The institutional review board at the Hospital Universitario Rio Hortega (reference: PI 138/20) approved the study protocol, which was conducted in accordance with the Declaration of Helsinki, and we followed the Strengthening the Reporting of Observational studies in Epidemiology (STROBE) statement [23]. The institutional review board granted a waiver on the obligation of collecting informed consent from participants. The study handled data from de-identified subjects; all patients were anonymized.

### 2.2. Population

From all calls for medical assistance to the 1-1-2 emergency number, participants with suspected COVID-19 infection and those transferred with high priority by ambulance to the corresponding ED were identified. Thereafter, patients aged between 18 and 80 with a positive SARS-CoV-2 test, as shown by polymerase chain reaction (PCR), were recruited for the study. 

The exclusion criteria included: patients under 18 or over 80 years, cases without analytical evidence of COVID-19 infection, patients with unknown comorbidities, and patients in whom it was not possible to collect parameters to calculate the analyzed indices.

### 2.3. Outcome

The primary outcome was the requirement for IMV. This outcome was adopted as an objective endpoint. In accordance with the recommendations of scientific societies [1,24,25], the upper cut-off age was set at 80 years, since these patients should preferably receive a high-concentration oxygen mask, high-flow oxygen therapy, or non-IMV of IMV. All IMV cases were re-checked by the head researcher.

### 2.4. Measures and Data Abstraction

Demographic covariates (age, sex, rural or urban area, and origin of nursery homes) were obtained from the standardized emergency medical services (EMS) medical records. Vital signs (respiratory rate, pulse oximetry saturation, systolic and diastolic arterial pressure, heart rate, and temperature) were recorded by an emergency registered nurse at the triage box upon initial access to the patient. Pulse oximetry saturation, systolic and diastolic arterial pressure, heart rate, and temperature were measured using the Connex^®^ Vital Signs Monitor (Welch Allyn Inc., Skaneateles Falls, NY, USA). Respiratory rate was calculated by counting respiratory cycles for 30 s. In cases of irregular or very shallow breathing, measurements were made by direct auscultation with a stethoscope. Glasgow coma scale and fraction of inspired oxygen whourere also recorded at the triage box.

Pulse oximetry saturation, fraction of inspired oxygen, and respiratory rate were used to calculate the SpO2/FiO2 ratio and the ROX index.

The following data were obtained by reviewing the electronic medical records: PCR test positive, hospital-inpatient and hospitalization time, ICU-admission date and stay period, IMV, in-hospital mortality, out-hospital mortality, aggregate mortality, and comorbidities to calculate the Charlson comorbidity index (CCI).

The hospital follow-up period for in-hospital patients lasted up to 120 days (patient of the cohort with the longest hospital stay).

### 2.5. Primary Data Analysis 

A database with anonymized records was specifically created for this research. Prior to analysis, the case registry was checked for logic and range, removing duplicate or ambiguous entries. None of the recorded variables had more than 5% missing values. Once all the parameters were entered into the database, scores were calculated using XLSTAT^®^ BioMED for Microsoft Excel^®^ version 14.4.0 software (Microsoft Inc., Redmond, WA, USA).

### 2.6. Statistical Analyses

Absolute values and percentages were used to represent categorical variable; for continuous variables, median and interquartile ranges (IQR) were used because they did not follow a normal distribution (normal distribution was assessed by Shapiro–Wilk test). The univariable comparison between each independent variable and the main outcome (IMV), or age range, was assessed by the Mann–Whitney U test or chi-squared test, when appropriate.

To compare both indices, the whole cohort was randomly split into training and validation subsets; the training sample was used to build the model and the validation subset was used to determine the predictive validity of each score. In particular, the discriminatory validity of the scales for the primary outcome was assessed by the area under the receiver operating characteristic (ROC) curve (AUC), calculating the confidence interval (95% CI) obtained by resampling (or bootstrapping) 2000 iterations for each case. Moreover, the specificity, sensitivity, positive predictive value, negative predictive value, positive likelihood ratio, and negative likelihood ratio were calculated for each case. With the objective of comparing ROCs, a Delong’s test was used. Finally, four groups of patients were derived from the following age ranges: <40, 41–55, 56–70, >70.

Data were analyzed using our own codes and base functions in R, version 4.0.3 (http://www.R-project.org; the R Foundation for Statistical Computing, Vienna, Austria).

## 3. Results

### 3.1. Cohort Overview

**Figure 1:**
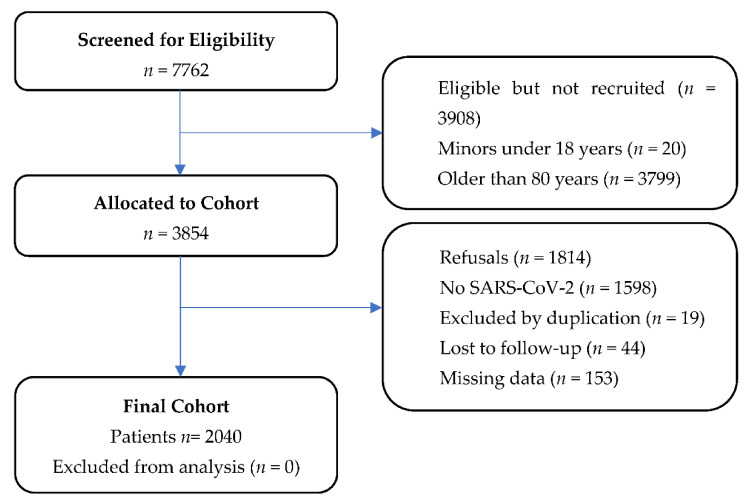
Flow chart of participants enrolled in the study. Abbreviations: SARS-CoV-2: severe acute respiratory syndrome coronavirus 2.

A total of 2040 patients were included in the study (Figure 1). The median age was 67 years (IQR: 55–75 years). Table 1 shows the global demographic characteristics and the comparison between IMV and non-IMV patients. The admission rate was 71.2% (1453 cases), with 10.1% (207 cases) of IMV. The in-hospital mortality rate was 35.3% (73 cases) in IMV vs. 9.7% (178 cases) of patients who did not require IMV. No differences in CCI between patients with or without IMV were found.

Table 2 shows the characteristics for each age range: <40, 41–55, 56–70, >70. Range #1 (patients under 40 years) represented 8.1% (165 cases) of the whole cohort; this group was characterized by a higher presence of females (59.4%), with negligible nursing home origin (only 1.8%), with both ICU-admission and IMV rates being extremely low (2.4%), without comorbidities, and with an associated mortality less than 1%. Range #2 (41–55 years, 18%, 367 cases) showed no differences in terms of sex, with a relatively low proportion of nursing home patients, a rate of ICU-admission and IMV of 8.4% and 6.8%, respectively, and with an aggregate mortality of 3.5% (13 cases). Range #3 (56–70 years, 33.7%, 687 cases) was mostly composed of males (61.39%), with 11.1% (76 cases) coming from nursing homes, with a mortality of 11.5% (79 cases), and with the highest ICU-admission and IMV rates of 13.4% and 13.2%, respectively. Finally, range #4 (older than 71 years, 40.2%, 821 cases) represented most of the cohort with the highest nursing home origin (23.8%), with ICU-admission and IMV rates of 11.9% and 10.6%, which are smaller than range #3; however, range #4 had the highest mortality rate in the cohort (26.3%). There is an inverse association between both the SpO2/FiO2 ratio and the ROX index and age, as both indexes decrease with increasing age; there is also a direct association between age and mortality (in-hospital and out-hospital), hospitalization time, and the presence of comorbidities.

### 3.2. SpO2/FiO2 Ratio and ROX Index Discrimination

**Figure 2:**
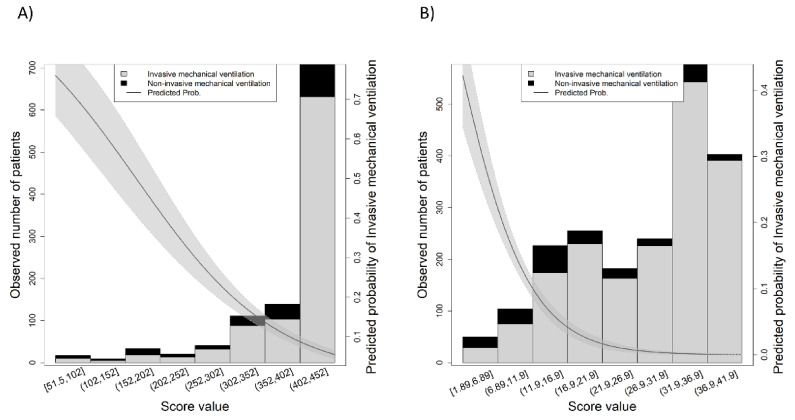
Observed number of cases for each of the scores: (**A**) SpO2/FiO2 ratio, (**B**) ROX index. The grey shadowed area shows the predicted probability of the outcome.

The discriminatory validity of the SpO2/FiO2 ratio was better than the ROX index, with an AUC = 0.801 (95% CI 0.746–0.855) and an AUC = 0.725 (95% CI 0.652–0.798), respectively. In fact, the direct comparison between AUCs yielded a significant difference (*p* = 0.001). Additionally, the observed number of cases for the value of each index is shown in Figure 2A for the case of SpO2/FiO2 ratio and Figure 2B for the case of ROX index, which also shows the predicted probability of IMV according to the value of the index. Supplementary Figure S1 showed both AUCs (SpO2/FiO2 ratio in grey and ROX index in black); further details of the discriminatory capacity can be found in the Supplementary Table S1.

### 3.3. SpO2/FiO2 Ratio and Age Association

**Figure 3:**
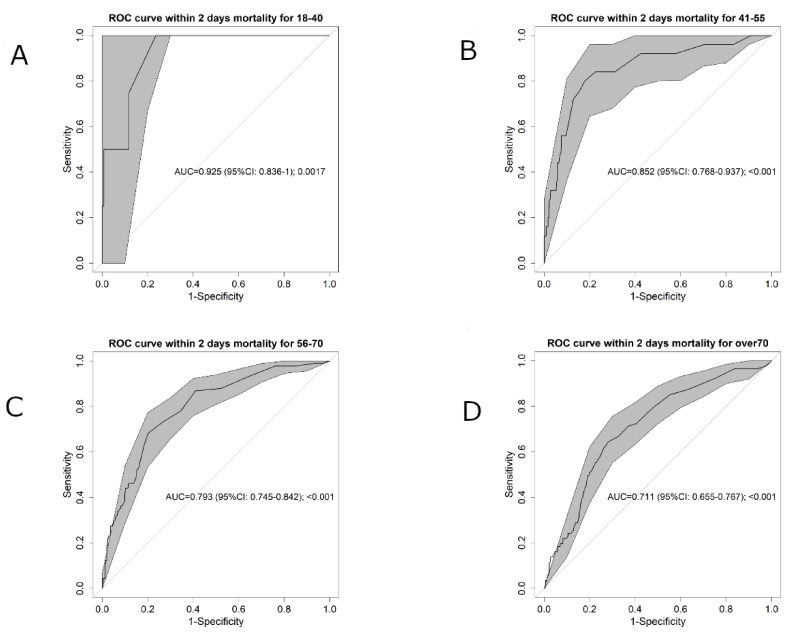
Area under the receiver operating characteristic (ROC) curve (AUC) for the four age ranges: (**A**) <40, (**B**) 41–55, (**C**) 56–70, (**D**) >70. The grey shadowed area shows the confidence interval (95% CI).

As the SpO2/FiO2 ratio performed better than the ROX index, the possible role of age on SpO2/FiO2 ratio performance was assessed by determining discriminatory capacity in the four age ranges. As shown in Figure 3, the AUC decreases with age, resulting in an AUC = 0.925 (95% CI 0.836–1) in the youngest group and an AUC = 0.711 (95% CI 0.655–0.767) in the oldest group. 

Lastly, using previous SpO2/FiO2 ratio cutoff points considered for the determination of mortality risk groups (18), we assessed the percentages of IMV for each group; the high-risk group (50–100) presented 44%, the intermediate group (101–426) presented 25%, and the low risk group (427–476) presented 5% of patients on IMV.

## 4. Discussion

To our knowledge, this is the first multicenter, derivation–validation, prognostic cohort study comparing the diagnostic accuracy of the SpO2/FiO2 ratio with the ROX index to predict IMV as a primary outcome in COVID-19 patients transferred by ambulance to an ED. In the current study, we found that the SpO2/FiO2 ratio had better accuracy than the ROX index in predicting IMV. 

In the final cohort, IMV rate was 10.1%, a percentage such as that observed in other studies (13%) [26]. Patient in-hospital mortality with IMV was 35.3%, a percentage slightly lower than the one already reported [26]; this is perhaps due to the lower percentage of elderly patients. IMV was higher for patients not living in nursing homes, in older patients (68 vs. 66 years, *p* < 0.001), and in women, in accordance with previous studies [27]. Interestingly, CCI was not a critical factor between IMV and non-IMV patients. 

The COVID-19 pandemic has created significant pressure on healthcare systems, and the burden of patient suffering has also affected the availability of resources such as ventilators [27]. Given this exceptional situation, consensus works from ethics groups of medical societies have been published, providing guidelines and recommendations to deal with resource shortages; these interventions assist decision-making criteria for the adoption of patient prioritization [1,24,25]. This certainly shows the necessity of a proper triage of patients to optimize available resources.

Different studies have shown that the early warning scores usually applied, such as the Modified Early Warning Score and Quick Sequential Organ Failure Assessment score, were inadequate to accurately predict respiratory failure in COVID-19 patients [28,29]. On the one hand, recent data indicated that the ROX index had moderate utility in predicting IMV in patients infected with COVID-19, especially in cases with moderate to acute respiratory failure [30]. Suliman et al. [12] showed that AUC value, as a predictor of IMV, on the first day of admission in non-ICU-conditions was 0.897; however, this was in a small study of 69 patients with pneumonia, so studies with larger sample sizes and patients with varying degrees of severity will be necessary to support these results. On the other hand, pre-pandemic studies showed that, in acute hypoxemic respiratory failure [31], the value of the SpO2/FiO2 ratio serves as control during noninvasive mechanical ventilation [32], or as a proxy measure for the calculation of the sepsis-related organ failure assessment score when partial pressure of oxygen in arterial blood is not available [33]; however, evidence related to COVID-19 is more limited. In this sense, Catoire et al. has suggested that the SpO2/FiO2 ratio is a reliable tool for hypoxemia screening in triage among patients admitted to the ED with respiratory symptoms [20]. Likewise, Lu et al. have shown that it could be used to improve the early adjustment of treatments—such as non-invasive or invasive ventilators, high-flow nasal cannula oxygen therapy, and extracorporeal membrane oxygenation—in the ICU [19]. Nonetheless, it has not yet been postulated as a clear predictor of orotracheal intubation. There are some novel models that predict, with similar AUCs as the one from SpO2/FiO2 ratio described here, the need for IMV in COVID-19 patients, but these scores require a blood sample that cannot be available immediately upon ED admission [34]. 

During the pandemic, a great deal of emphasis has been placed on so-called *silent hypoxemia,* which is characterized by a dissociation between SpO_2_ and ventilatory dynamics [35,36]. The ventilatory response to hypoxemia is highly variable and is usually observed oin people who practically do not increase their ventilation when their oxygen decreases [7,8,37]. This unique pathophysiology of the disease could explain a decrease in the diagnostic accuracy of the ROX index when compared to SpO2/FiO2, though this could also be affected by an underestimated/incorrect evaluation of respiratory rates.

Assessment of the oxygenation status of critical patients is of utmost importance to plan interventions and prognosis. We demonstrated the prognostic value of SpO2/FiO2 in COVID-19 patients where its decreasing trajectory was directly associated with age and an increased risk of deterioration and mortality. These results support those published by Lu et al. and Catoire et al. [19,20].

A variable of particular interest to consider when interpreting the SpO2/FiO2 ratio is age [38,39]. The SpO2/FiO2 ratio is more clearly predicted in younger patients (<40 years); a reason for this could be found by the fact that pre-existing co-morbidities may not represent a limitation for orotracheal intubation, which translates into a higher correlation between the ratio value and the requirement of IMV. The index should be used with greater caution in the group over 70 years old.

To determine to what extent the ratio could be useful as a tool to predict outcomes, we have chosen to classify the patients into three groups, with SpO2/FiO2 cut-off points previously considered for the determination of mortality risk groups [18]. Our results show that we must carry out a closer study of intermediate (SpO2/FiO2 101–426) and high-risk (SpO2/FiO2 50–100) patients, as these groups represent an increased requirement for IMV (25% and 44%, respectively) as compared to the low-risk group (SpO2/FiO2: 427–476, with 5% of patients with IMV). 

Avoiding emerging procedures during the COVID-19 pandemic is important due to the risk of viral transmission to health personnel [28]. Therefore, a highly specific model can avoid unnecessary intubation [40]. This lends even more value to the performance of the SpO2/FiO2 ratio, since we obtained a specificity of 89.49 (95% CI: 87.65–91.32). These data reaffirm the predictive capacity of the SpO2/FiO2 ratio, a crucial piece of information during the extreme phases of the current pandemic, when it was necessary, on numerous occasions, to select the order of intubation or, in the worst scenario, to decide which patients most fulfilled the criteria for IMV. SpO2/FiO2 can help in the critical decision-making process, representing a straightforward tool to implement in the system that can clarify required interventions.

## 5. Limitations 

Our investigation is not free of limitations. First, SARS-CoV-2 virological status in our study is based on the presence of a positive PCR. The number of false positives is likely to be small, but we should assume the existence of false negatives. Second, because the standardization of intubation was not decided a priori, one could argue that the outcome of mechanical ventilation was somewhat subjective, which could be a function of local practices; since all eight hospitals cooperated in previous common studies, the guidelines for airway and ventilation management were similar. Third, the population sample collected during this period cannot be considered representative of a standard sample of patients transferred by the EMS due to the very character of the pandemic. Likewise, a patient selection bias may be present in this manuscript due to non-probability sampling methods. To minimize bias, the study involved units working in urban and rural areas all over 24 h and 7 days a week. Fourth, the data extractors were not blind. To ensure that the results were not a matter of interpretation, a two-step verification between an associate investigator from each hospital and the principal investigator was performed in those cases requiring invasive mechanical ventilation. Previously validated indices were used to minimize possible interobserver bias among researchers. Finally, it should be noted that we are dealing with a retrospective study, with the methodological drawbacks that this may entail. As soon as the pandemic allows health systems to work in an ordinary way, it is necessary to carry out prospective studies to determine the true nature of the biomarkers analyzed here.

## 6. Conclusions

The current study suggested that the SpO2/FiO2 ratio is a simple, non-invasive, and promising tool for predicting the risk of IMV in patients infected with COVID-19. It can also be performed in an ED. The timely identification of these cases could help to improve survival rates, together with the reasonable and appropriate cost-effective allocation of resources. The use of the SpO2/FiO2 ratio can help in performing an early estimation of the degree of hypoxemia in patients infected with COVID-19, even in patients who are seriously ill or at high risk of clinical deterioration, but with a low initial suspicion of infection; thus, overall survival might be improved. 

In summary, the SpO2/FiO2 ratio presents a good combination of precision, non-invasiveness, and speed, all of which are especially useful in scenarios in which a blood gas analyzer is not available. This could help EMS personnel to assess clinical severity in complex decision-making processes during ED procedures.

## Figures and Tables

**Table 1:** Baseline patient characteristics based on invasive mechanical ventilation requirements.

Variable ^1^	Total	IMV	Non-IMV	*p* Value ^2^
No. (%) with data	2040 (100)	207 (10.1)	1833 (89.9)	NA
Age, year	67 (55–75)	68 (62–75)	66 (54–75)	<0.001
Age, group, year				
<40	165 (8.1)	4 (1.9)	161 (8.8)	
41–55	367 (18)	25 (12.1)	342 (18.7)	0.003
56–70	687 (33.7)	91 (44)	596 (32.5)	0.041
>71	821 (40.2)	87 (42)	734 (40)	0.113
Sex				
Female	883 (43.3)	152 (73.4)	828 (45.2)	
Male	1157 (56.7)	55 (22.6)	1005 (54.8)	<0.001
Nursing homes	288 (14.1)	6 (2.9)	282 (5.4)	<0.001
Zone, urban	1117 (54.1)	113 (54.9)	1004 (54.8)	0.960
Triage ED evaluation				
Respiratory rate, bpm	14 (12–24)	24 (13–28)	14 (12–21)	<0.001
Pulse oximetry saturation, %	95 (91–97)	89 (83–93)	95 (82–97)	<0.001
Fraction of inspired oxygen, %	0.21 (0.21–0.21)	0.21 (0.21–0.24)	0.21 (0.21–0.21)	<0.001
Systolic arterial pressure, mmHg	126 (113–143)	122 (110−139)	126 (113–141)	0.047
Diastolic arterial pressure, mmHg	76 (67–84)	72 (64–82)	76 (68–84)	0.009
Heart rate, bpm	90 (78–101)	90 (79–105)	90 (78–101)	0.482
Temperature, °C	36.7 (36.2–37.3)	37 (36.2–37.8)	36.7 (36.2–37.3)	0.006
Glasgow coma scale, point	15 (15–15)	15 (15–15)	15 (15–15)	0.421
Outcomes				
SpO2/FiO2 ratio	448 (424–462)	405 (319–438)	452 (429–462)	<0.001
ROX index	31.3 (18.2–35.9)	16.9 (11.9–30.9)	32.1 (19.5–36.2)	<0.001
Hospital-inpatient	1453 (71.2)	207 (100)	1246 (68)	<0.001
Hospitalization time, day	9 (5–16)	26 (14–46)	8 (5–13)	<0.001
Intensive care unit-admission	225 (11)	197 (95.2)	28 (1.5)	<0.001
Intensive care unit time, day	16 (7–30)	15 (7–30)	10 (5–64)	<0.001
In-hospital mortality	251 (12.3)	73 (35.3)	178 (9.7)	<0.001
Out-hospital mortality	58 (2.8)	7 (3.4)	51 (2.8)	0.623
Aggregate mortality	309 (15.1)	80 (38.6)	229 (12.5)	<0.001
CCI, point	1 (0–2)	1 (0–2)	1 (0–2)	0.733
AIDS	3 (0.1)	0	3 (0.2)	NA
Solid tumor metastatic	30 (1.5)	2 (1)	28 (1.5)	0.525
Liver disease severe	54 (2.6)	4 (1.9)	50 (2.7)	0.499
Lymphoma	11 (0.5)	0	11 (0.6)	NA
Leukemia	22 (1.1)	2 (1)	20 (1.1)	0.869
Solid tumor localized	233 (11.4)	22 (10.6)	211 (11.5)	0.705
DM end organ damage	88 (4.3)	14 (6.8)	74 (4)	0.067
Severe chronic kidney disease	168 (8.2)	21 (10.1)	147 (8)	0.292
Hemiplegia	48 (2.4)	1 (0.5)	47 (2.6)	0.061
DM uncomplicated	345 (16.9)	42 (20.3)	303 (16.5)	0.172
Liver disease mild	63 (3.1)	8 (3.9)	55 (3)	0.496
Peptic ulcer disease	54 (2.6)	9 (4.3)	45 (2.5)	0.108
Connective disease	69 (3.3)	7 (3.4)	61 (3.3)	0.967
COPD	174 (8.5)	20 (9.7)	154 (8.4)	0.539
Dementia	154 (7.5)	1 (0.5)	153 (8.3)	<0.001
Cerebrovascular disease	112 (5.5)	11 (5.3)	101 (5.5)	0.907
Peripheral vascular disease	140 (6.9)	22 (10.6)	118 (6.4)	0.024
Congestive heart failure	107 (5.2)	14 (6.8)	93 (5.1)	0.302
Myocardial infarction	136 (6.7)	20 (9.7)	116 (6.3)	0.068

Abbreviations: ED: emergency department; SpO2/FiO2 ratio: pulse oximetry saturation/fraction of inspired oxygen ratio; ROX index: ratio of oxygen saturation, as measured by pulse oximetry/FiO2 to respiratory rate; NA: not applicable; CCI: Charlson comorbidity index; AIDS: acquired immunodeficiency syndrome; DM: diabetes mellitus; COPD: chronic obstructive pulmonary disease; PVD: peripheral vascular disease. ^1^ Values expressed as total number (fraction) and medians [25 percentile–75 percentile], as appropriate. ^2^ The Mann–Whitney U test or chi-squared test was used as appropriate.

**Table 2:** Population distribution based by age categories under analysis.

Variable ^1^	<40	41–55	56–70	>70	*p* Value ^2^
No. (%) with data	165 (8.1)	367 (18)	687 (33.7)	821 (40.2)	NA
Age, y	32 (26–38)	50 (45–53)	64 (60–67)	76 (73–78)	NA
Sex, female					
Female	98 (59.4)	176 (48)	262 (38.1)	346 (42.3)	<0.001
Male	67 (40.6)	191 (52)	425 (61.9)	474 (57.7)	<0.001
Nursing homes	3 (1.8)	14 (3.8)	76 (11.1)	195 (23.8)	<0.001
Zone, urban	99 (60)	188 (51.2)	378 (55)	452 (55.1)	0.293
Triage ED evaluation					
Respiratory rate, bpm	13 (12–15)	14 (12–20)	14 (12–24)	14 (13–25)	<0.001
Pulse oximetry saturation, %	97 (95–99)	96 (93–98)	94 (91–96)	93 (89–96)	<0.001
FiO2, %	0.21 (0.21–0.21)	0.21 (0.21–0.21)	0.21 (0.21–0.21)	0.21 (0.21–0.21)	<0.001
SAP, mmHg	120 (111–134)	125 (114–138)	127 (115–143)	127 (111–144)	0.008
DAP, mmHg	77 (70–85)	80 (71–88)	77 (68–84)	72 (64–80)	<0.001
Heart rate, bpm	94 (82–106)	92 (81–103)	90 (80–100)	87 (75–101)	<0.001
Temperature, °C	36.6 (36.2–37.1)	36.8 (36.3–37.3)	36.7 (36.2–37.4)	36.6 (36.2–37.3)	0.149
Glasgow coma scale, point	15 (15–15)	15 (15–15)	15 (15–15)	15 (15–15)	0.233
Outcomes					
SpO2/FiO2 ratio	462 (452–471)	457 (443–467)	448 (424–457)	438 (405–452)	<0.001
ROX index	35.3 (30.3–38.2)	32.6 (22.2–36.6)	31.2 (18.3–35.9)	28.8 (16.8–34.8)	<0.001
IMV	4 (2.4)	25 (6.8)	91 (13.2)	87 (10.6)	<0.001
Hospital-inpatient	54 (32.7)	203 (55.3)	518 (75.4)	678 (82.6)	<0.001
Hospitalization time, day	6 (3–10)	8 (5–13)	9 (6–16)	9 (5–17)	<0.001
Intensive care unit-admission	4 (2.4)	31 (8.4)	92 (13.4)	98 (11.9)	<0.001
Intensive care unit time, day	14 (4–31)	7 (4–15)	15 (7–28)	22 (11–40)	<0.001
In-hospital mortality	1 (0.6)	10 (2.7)	61 (8.9)	179 (21.8)	<0.001
Out-hospital mortality	0	3 (0.8)	18 (2.6)	37 (4.5)	<0.001
Aggregate mortality	1 (0.6)	13 (3.5)	79 (11.5)	216 (26.3)	<0.001
CCI, point	0 (0–0)	0 (0–1)	1 (0–2)	1 (0–3)	<0.001

Abbreviations: ED: emergency department; FiO2: fraction of inspired oxygen; SAP: systolic arterial pressure; DAP: diastolic arterial pressure; SpO2/FiO2 ratio: pulse oximetry saturation/fraction of inspired oxygen ratio; ROX index: ratio of oxygen saturation, as measured by pulse oximetry/FiO2 to respiratory rate; IMV: invasive mechanical ventilation; NA: not applicable; CCI: Charlson comorbidity index. ^1^ Values expressed as total number (fraction) and medians [25 percentile–75 percentile], as appropriate. ^2^ The Mann–Whitney U test or chi-squared test was used as appropriate.

## Data Availability

The data of the study are available to other researchers upon reasonable request from the corresponding author.

## Supplementary Materials

The following are available online at https://www.mdpi.com/article/10.3390/biomedicines9081036/s1, Figure S1: Area under the receiver operating characteristic (ROC) curve (AUC) for SpO2/FiO2 ratio in grey and ROX index in black, Table S1: Further details of the predictive capacity of SpO2/FiO2 ratio and ROX index.

## References

[1] Rubio O., Estella A., Cabre L., Saralegui-Reta I., Martin M.C., Zapata L., Esquerda M., Ferrer R., Castellanos A., Trenado J. (2020). Ethical recommendations for a difficult decision-making in intensive care units due to the exceptional situation of crisis by the COVID-19 pandemia: A rapid review & consensus of experts. Med. Intensiv..

[2] Escudero-Acha P., Leizaola O., Lázaro N., Cordero M., Gomez-Acebo I., González-Castro A. (2020). Age as a limiting factor of admission to an intensive care unit. Med. Intensiv..

[3] Griffin K.M., Karas M.G., Ivascu N.S., Lief L. (2020). Hospital Preparedness for COVID-19: A Practical Guide from a Critical Care Perspective. Am. J. Respir. Crit. Care Med..

[4] Lentz T., Groizard C., Colomes A., Ozguler A., Baer M., Loeb T. (2021). Collective Critical Care Ambulance: An innovative transportation of critical care patients by bus in COVID-19 pandemic response. Scand. J. Trauma Resusc. Emerg. Med..

[5] Ferron R., Agarwal G., Cooper R., Munkley D. (2021). The effect of COVID-19 on emergency medical service call volumes and patient acuity: A cross-sectional study in Niagara, Ontario. BMC Emerg. Med..

[6] Grasselli G., Greco M., Zanella A., Albano G., Antonelli M., Bellani G., Bonanomi E., Cabrini L., Carlesso E., Castelli G. (2020). Risk Factors Associated with Mortality among Patients with COVID-19 in Intensive Care Units in Lombardy, Italy. JAMA Intern. Med..

[7] Gibson P.G., Qin L., Puah S.H. (2020). COVID-19 acute respiratory distress syndrome (ARDS): Clinical features and differences from typical pre-COVID-19 ARDS. Med. J. Aust..

[8] Gattinoni L., Chiumello D., Rossi S. (2020). COVID-19 pneumonia: ARDS or not?. Crit. Care.

[9] De Vita N., Scotti L., Cammarota G., Racca F., Pissaia C., Maestrone C., Colombo D., Olivieri C., Della Corte F., Barone-Adesi F. (2021). Predictors of intubation in COVID-19 patients treated with out-of-ICU continuous positive airway pressure. Pulmonology.

[10] Gianstefani A., Farina G., Salvatore V., Alvau F., Artesiani M.L., Bonfatti S., Campinoti F., Caramella I., Ciordinik M., Lorusso A. (2021). Role of ROX index in the first assessment of COVID-19 patients in the emergency department. Intern. Emerg. Med..

[11] Goh K.J., Chai H.Z., Ong T.H., Sewa D.W., Phua G.C., Tan Q.L. (2020). Early prediction of high flow nasal cannula therapy outcomes using a modified ROX index incorporating heart rate. J. Intensiv. Care.

[12] Suliman L.A., Abdelgawad T.T., Farrag N.S., Abdelwahab H.W. (2021). Validity of ROX index in prediction of risk of intubation in patients with COVID-19 pneumonia. Adv. Respir. Med..

[13] Marraro G.A., Li Z., Piga M.A. (2018). Searching for Biomarkers With Predictive Value in Pediatric Acute Lung Injury: Can SpO2/FIO2 Be Used Instead of PaO2/FIO2 as an Index to Predict Outcome?. Pediatr. Crit. Care Med..

[14] Roca O., Caralt B., Messika J., Samper M., Sztrymf B., Hernández G., García-de-Acilu M., Frat J.-P., Masclans J.R., Ricard J.-D. (2019). An Index Combining Respiratory Rate and Oxygenation to Predict Outcome of Nasal High-Flow Therapy. Am. J. Respir. Crit. Care Med..

[15] Prower E., Grant D., Bisquera A., Breen C.P., Camporota L., Gavrilovski M., Ponting M., Douirih A., Gloveri G.W. (2021). The ROX index has greater predictive validity than NEWS2 for deterioration in Covid-19. EClinicalMedicine.

[16] Fink D.L., Goldman N.R., Cai J., El-Shakankery K.H., Sismey G.E., Gupta-Wright A., Tai C.X. (2021). ROX Index to Guide Management of COVID-19 Pneumonia. Ann. Am. Thorac. Soc.

[17] Brown S.M., Grissom C.K., Moss M., Rice T.W., Schoenfeld D., Hou P.C., Thompson B.T., Brower R.G., NIH/NHLBI PETAL Network Collaborators (2016). Nonlinear Imputation of Pao2/Fio2 from Spo2/Fio2 among Patients with Acute Respiratory Distress Syndrome. Chest.

[18] Martín-Rodríguez F., López-Izquierdo R., del Pozo Vegas C., Delgado-Benito J.F., Ortega G.J., Villamor M.A.C., Sanz-García A. (2021). Association of Prehospital Oxygen Saturation to Inspired Oxygen Ratio with 1-, 2-, and 7-Day Mortality. JAMA Netw. Open.

[19] Lu X., Jiang L., Chen T., Wang Y., Zhang B., Hong Y. (2020). Continuously available ratio of SpO/FiO serves as a noninvasive prognostic marker for intensive care patients with COVID-19. Respir. Res..

[20] Catoire P., Tellier E., de la Rivière C., Beauvieux M.C., Valdenaire G., Galinski M., Revel P., Combes X., Gil-Jardiné C. (2021). Assessment of the SpO/FiO ratio as a tool for hypoxemia screening in the emergency department. Am. J. Emerg. Med..

[21] Lemiale V., Dumas G., Demoule A., Pène F., Kouatchet A., Bisbal M., Nseir S., Argaud L., Kontar L., Klouche K. (2021). Performance of the ROX index to predict intubation in immunocompromised patients receiving high-flow nasal cannula for acute respiratory failure. Ann. Intensiv. Care.

[22] Vega M.L., Dongilli R., Olaizola G., Colaianni N., Sayat M.C., Pisani L., Romagnoli M., Spoladore G., Prediletto I., Montiel G. (2021). COVID-19 Pneumonia and ROX index: Time to set a new threshold for patients admitted outside the ICU. Pulmonology.

[23] von Elm E., Altman D.G., Egger M., Pocock S.J., Gøtzsche P.C., Vandenbroucke J.P. (2007). The Strengthening the Reporting of Observational Studies in Epidemiology (STROBE) statement: Guidelines for reporting observational studies. Lancet.

[24] Cook T.M., El-Boghdadly K., McGuire B., McNarry A.F., Patel A., Higgs A. (2020). Consensus guidelines for managing the airway in patients with COVID-19: Guidelines from the Difficult Airway Society, the Association of Anaesthetists the Intensive Care Society, the Faculty of Intensive Care Medicine and the Royal College of Anaesthetist. Anaesthesia.

[25] Leclerc T., Donat N., Donat A., Pasquier P., Libert N., Schaeffer E., D’Aranda E., Cotte J., Fontaine B., Perrigault P.-F. (2020). Prioritisation of ICU treatments for critically ill patients in a COVID-19 pandemic with scarce resources. Anaesth. Crit. Care Pain Med..

[26] Karagiannidis C., Mostert C., Hentschker C., Voshaar T., Malzahn J., Schillinger G., Klauber J., Janssens U., Marx G., Weber-Carstens S. (2020). Case characteristics, resource use, and outcomes of 10,021 patients with COVID-19 admitted to 920 German hospitals: An observational study. Lancet Respir. Med..

[27] Emanuel E.J., Persad G., Upshur R., Thome B., Parker M., Glickman A., Zhang C., Boyle C., Smith M., Phillips J.P. (2020). Fair Allocation of Scarce Medical Resources in the Time of Covid-19. N. Engl. J. Med..

[28] Shashikumar S.P., Wardi G., Paul P., Carlile M., Brenner L.N., Hibbert K.A., North C.M., Mukerji S.S., Robbins G.K., Shao Y.-P. (2021). Development and Prospective Validation of a Deep Learning Algorithm for Predicting Need for Mechanical Ventilation. Chest.

[29] Xie J., Tong Z., Guan X., Du B., Qiu H., Slutsky A.S. (2020). Critical care crisis and some recommendations during the COVID-19 epidemic in China. Intensiv. Care Med..

[30] Zucman N., Mullaert J., Roux D., Roca O., Ricard J.D. (2020). Prediction of outcome of nasal high flow use during COVID-19-related acute hypoxemic respiratory failure. Intensiv. Care Med..

[31] Chen W.L., Lin W.T., Kung S.C., Lai C.C., Chao C.M. (2018). The value of oxygenation saturation index in predicting the outcomes of patients with acute respiratory distress syndrome. J. Clin. Med..

[32] Frat J.P., Marie D., Thille A.W. (2019). Acute respiratory failure:non intubation assist methods for the acutely deteriorating patient. Curr. Opin. Crit. Care.

[33] Seymour C.W., Liu V.X., Iwashyna T.J., Brunkhorst F.M., Rea T.D., Scherag A., Rubenfeld G., Kahn J.M., Shankar-Hari M., Singer M. (2016). Assessment of clinical criteria for sepsis: For the third international consensus definitions for sepsis and septic shock (sepsis-3). JAMA.

[34] García-Gordillo J.A., Camiro-Zúñiga A., Aguilar-Soto M., Cuenca D., Cadena-Fernández A., Khouri L.S., Rayek J.N., Mercado M., The ARMII Study Group (2021). COVID-IRS: A novel predictive score for risk of invasive mechanical ventilation in patients with COVID-19. PLoS ONE.

[35] Tobin M.J., Laghi F., Jubran A. (2020). Why COVID-19 Silent Hypoxemia Is Baffling to Physicians. Am. J. Respir. Crit. Care Med..

[36] Bickler P.E., Feiner J.R., Lipnick M.S., McKleroy W. (2021). “Silent” Presentation of Hypoxemia and Cardiorespiratory Compensation in COVID-19. Anesthesiology.

[37] Quaresima V., Ferrari M. (2020). COVID-19: Efficacy of prehospital pulse oximetry for early detection of silent hypoxemia. Crit. Care.

[38] Fang X., Li S., Yu H., Wang P., Zhang Y., Chen Z., Li Y., Cheng L., Li W., Jia H. (2020). Epidemiological, comorbidity factors with severity and prognosis of COVID-19: A systematic review and meta-analysis. Aging.

[39] Chen Y., Klein S.L., Garibaldi B.T., Li H., Wu C., Osevala N.M., Li T., Margolick J.B., Pawelec G., Leng S.X. (2021). Aging in COVID-19: Vulnerability, immunity and intervention. Ageing Res. Rev..

[40] Meng L., Qiu H., Wan L., Ai Y., Xue Z., Guo Q., Deshpande R., Zhang L., Meng J., Tong C. (2020). Intubation and Ventilation amid the COVID-19 Outbreak: Wuhan’s Experience. Anesthesiology.

