# Supplementary material for: Role of SpO2/FiO2 Ratio and ROX Index in Predicting Early Invasive Mechanical Ventilation in COVID-19. A Pragmatic, Retrospective, Multi-Center Study
Source: Biomedicines. 2021 Aug 18;9(8):1036. doi: 10.3390/biomedicines9081036 (PMC8392288; doi:10.3390/biomedicines9081036)
Supplement: Supplementary file 1 [file biomedicines-09-01036-s001.zip › biomedicines-1333618-supplementary.pdf]

Supplementary material

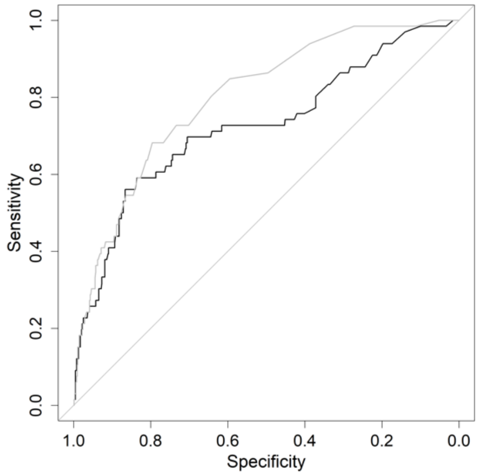

Supplementary Figure S1. Area under the receiver operating characteristic (ROC) curve (AUC) for SpO2/FiO2 ratio in grey and ROX index in black.

**SpO2/FiO2 ratio**	specificity	sensitivity	positive predictive value	negative predictive value	positive likelihood ratio	negative likelihood ratio
Estimate	89,49	26,80	34,10	92,01	4,92	0,77
Lower bound	87,65	24,24	33,04	91,79	4,73	0,75
Upper bpund	91,32	29,36	35,16	92,22	5,12	0,80
Standard deviation	0,93	1,30	0,54	0,11	0,10	0,01
**ROX index**	specificity	sensitivity	positive predictive value	negative predictive value	positive likelihood ratio	negative likelihood ratio
Estimate	29,79	79,80	15,86	97,38	1,86	0,25
Lower bound	21,63	73,34	13,80	96,66	1,52	0,18
Upper bpund	37,94	86,26	17,93	98,10	2,19	0,32
Standard deviation	4,11	3,25	1,04	0,36	0,17	0,03

Supplementary Table S1. Further details of the predictive capacity of SpO2/FiO2 ratio and ROX index
